# Accessory articulation of the cervical vertebrae transverse processes simulating a fracture: Incidental findings of an uncommon anatomical variant

**DOI:** 10.1259/bjrcr.20210012

**Published:** 2021-06-10

**Authors:** Natasha Naidoo, Nausheen Khan

**Affiliations:** 1 Department of Radiology, University of Pretoria, Pretoria, South Africa

## Abstract

Accessory articulation of the transverse processes of the cervical vertebrae is an extremely rare congenital anomaly. We present two cases of accessory articulation of the transverse processes of the left C5/C6 and C6/C7 cervical vertebrae. The articulation at C5/C6 was found in a 34-year-old male following a mob assault, the C6/C7 accessory articulation occurred in a 28-year-old female involved in a high velocity motor-vehicle accident. Cervical spine fractures were suspected in both cases. Recognition of this variant anatomy and differentiation from a fracture is important especially in an acute trauma setting to prevent unnecessary immobilization and inappropriate specialist referral of these patients. Our search revealed only five previously reported cases in the literature mostly occurring at the C5/C6 level. This is only the second case ever described at the C6/C7 level.

## Case series

### Case 1

A 34-year-old male presented with multiple facial fractures following a mob-assault. Computerized Tomography (CT) scan was performed using a helical multi-detector 16-slice CT scanner (Phillips Brilliance Netherlands) to further assess the facial fractures and to exclude associated brain and cervical spine injuries. Reformatted images were obtained in the axial, coronal and sagittal planes. A left C5/C6 transverse process articulation was found incidentally.

### Case 2

A 28-year-old female with no known medical co-morbidities presented to our emergency room following a high velocity motor-vehicle accident. She was an unrestrained driver with a Glasgow Coma Scale (GCS) of 14/15 who reported cervical spine tenderness. A CT scan was done in accordance with the Canadian CT head rules. A helical 16-slice multi-detector CT scanner (Phillips Brilliance Netherlands) was used with reformatted images obtained in the axial, coronal and sagittal planes. The patient was found to have mild traumatic brain injury with an uncommon left C6/C7 transverse process accessory articulation mimicking a fracture. No acute fractures or dislocations of the cervical spine were demonstrated.

## Discussion

Accessory articulation of the transverse processes of the cervical vertebrae is an extremely rare congenital anomaly that was first described in the 1960s.^1^ Our search revealed five previously reported cases in the literature that mainly occurred at the C5/C6 articulation.^2–6^ This is only the second case documented at the C6/C7 level.^2^

**Figure 1.**
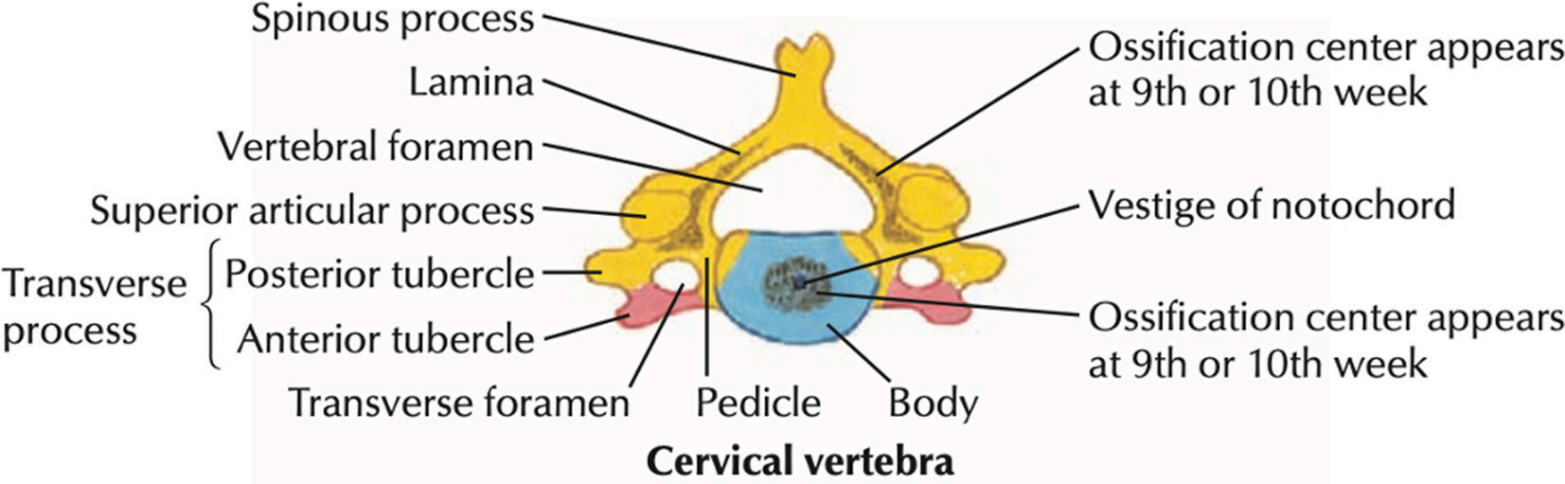
Primary ossification centres of the cervical vertebrae.^7^

The anomaly can be explained through embryological developments in the cervical spine. Each cervical vertebra has three ossification centers: a centrum and two vertebral neural arches. The transverse processes are formed by the lateral extension of the centres of the neural arches (Figure 1).^7^ A separate ossification center may develop in the sixth fetal month from which the anterior costal transverse process arises. By 6 years of age, it fuses with the main ossification centres of the transverse process. The costal portion of the transverse process may enlarge and elongate the anterior tubercle, giving rise to an unusual variant, an accessory articulation of the transverse processes, which is a form of the more common supernumery cervical rib.^2–4,6–9^

**Figure 2.**
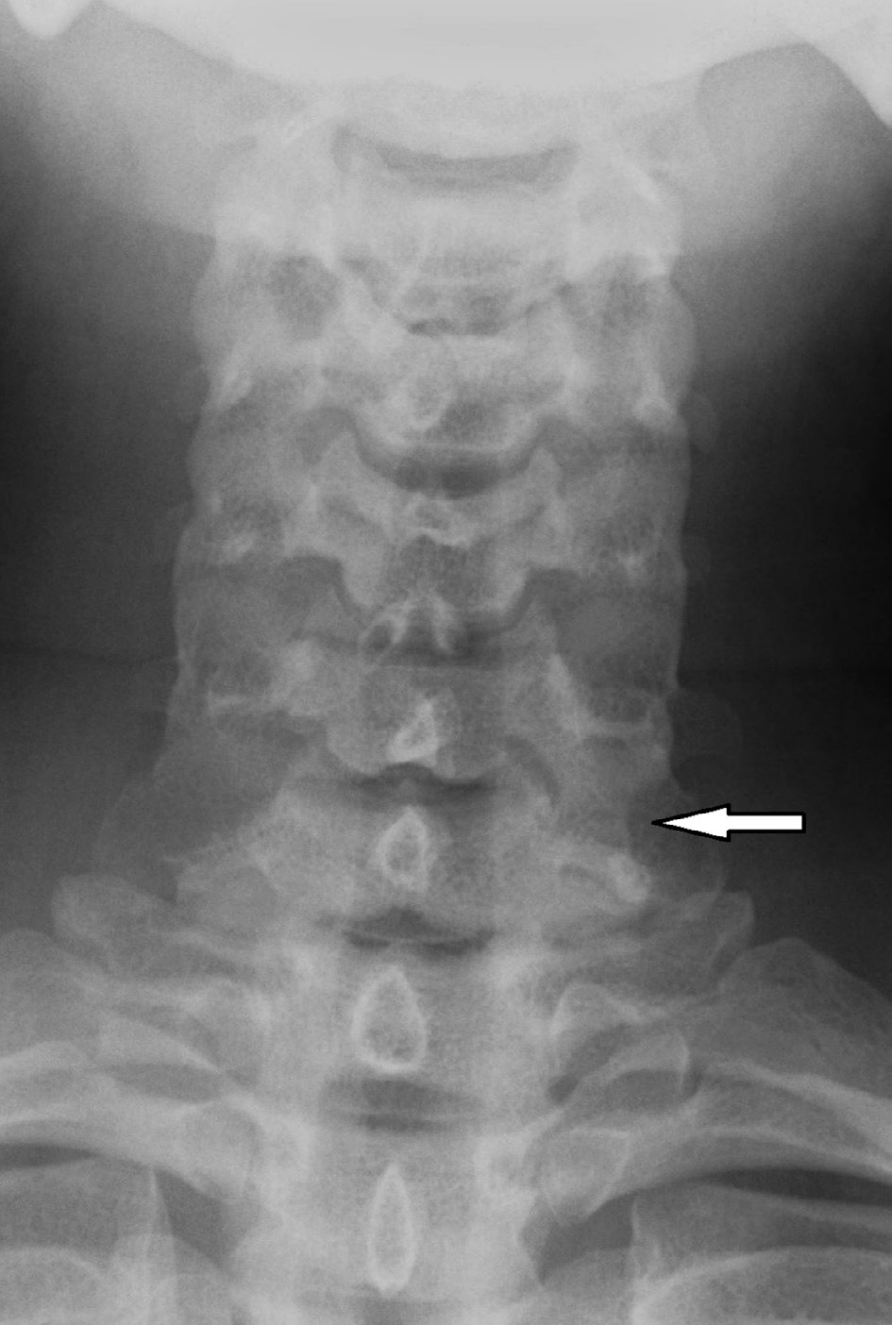
Anteroposterior radiograph of the cervical spine in a 28-year-old female following a motor vehicle accident. It shows a bony projection extending anterior to the C6/C7 vertebral bodies with a radiolucent line between the lateral masses.

**Figure 3.**
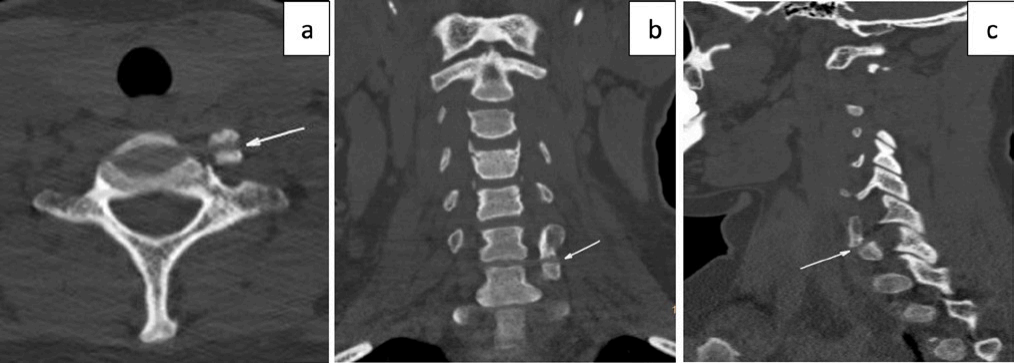
Cervical CT performed in the same patient with axial, coronal and sagittal reformations shows left-sided elongation of the anterior tubercle of the C6/C7 transverse process with an accessory articulation mimicking a fracture.

**Figure 4.**
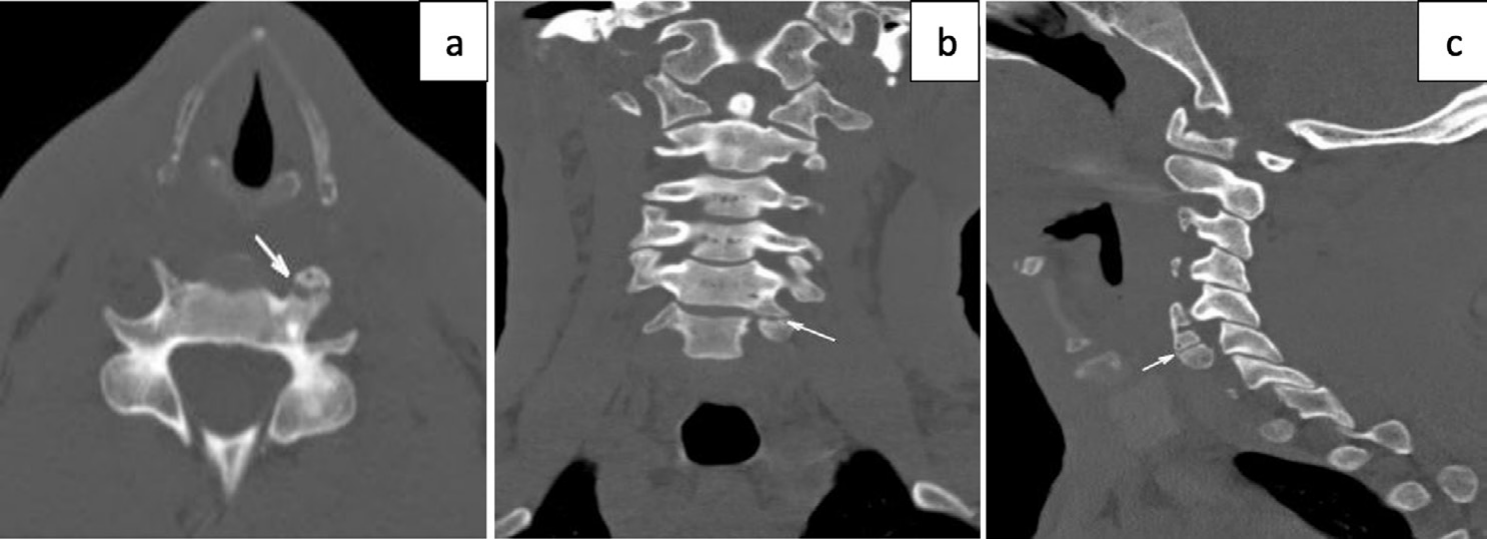
34-year-old male following a mob-assault. Cervical CT with axial, coronal and sagittal reformations shows an incidental left-sided elongation of the anterior tubercle of the C5/C6 transverse process with an accessory articulation.

**Figure 5.**
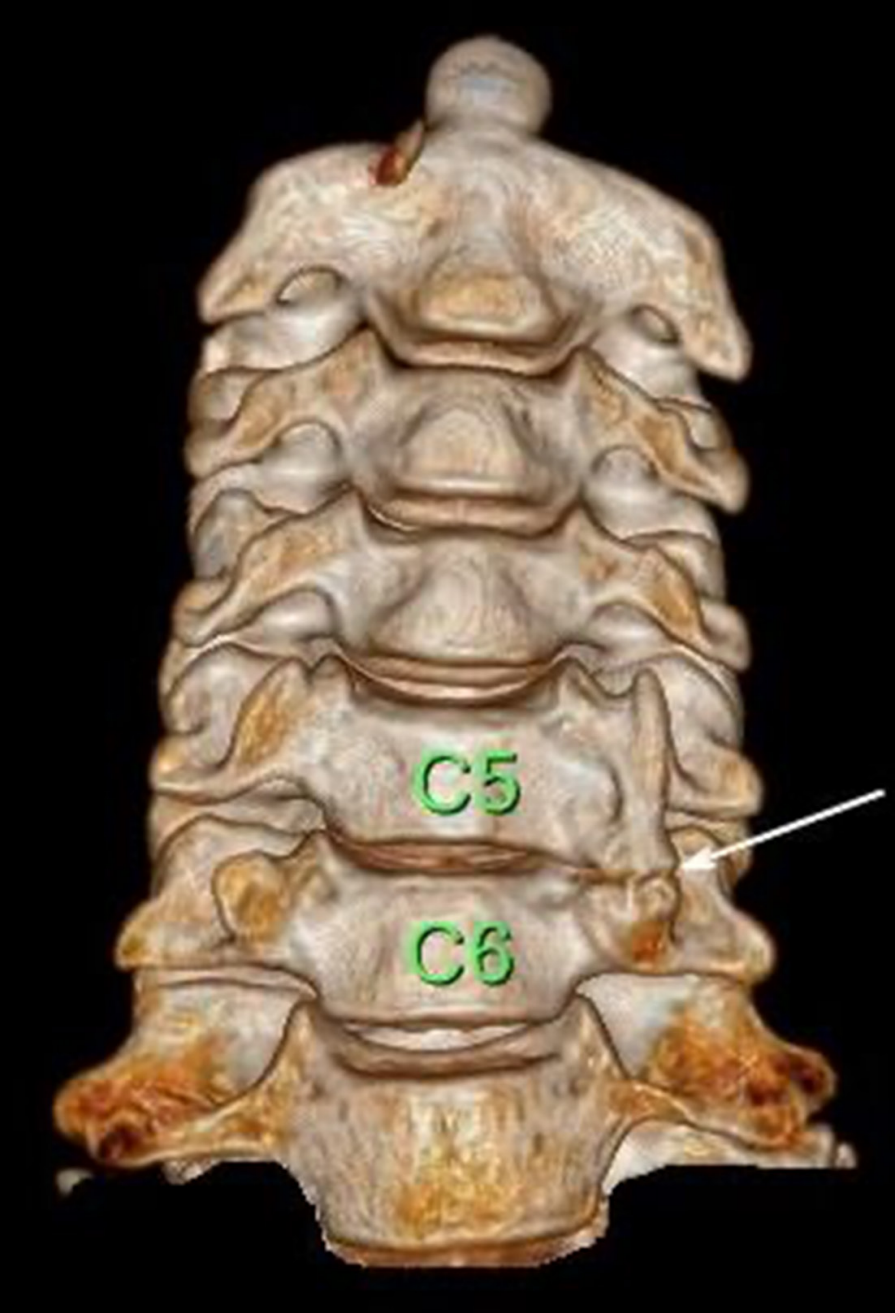
Coronal volume-rendered CT images clearly show the accessory articulation between the C5/C6 transverse processes without any associated fractures.

**Figure 6.**
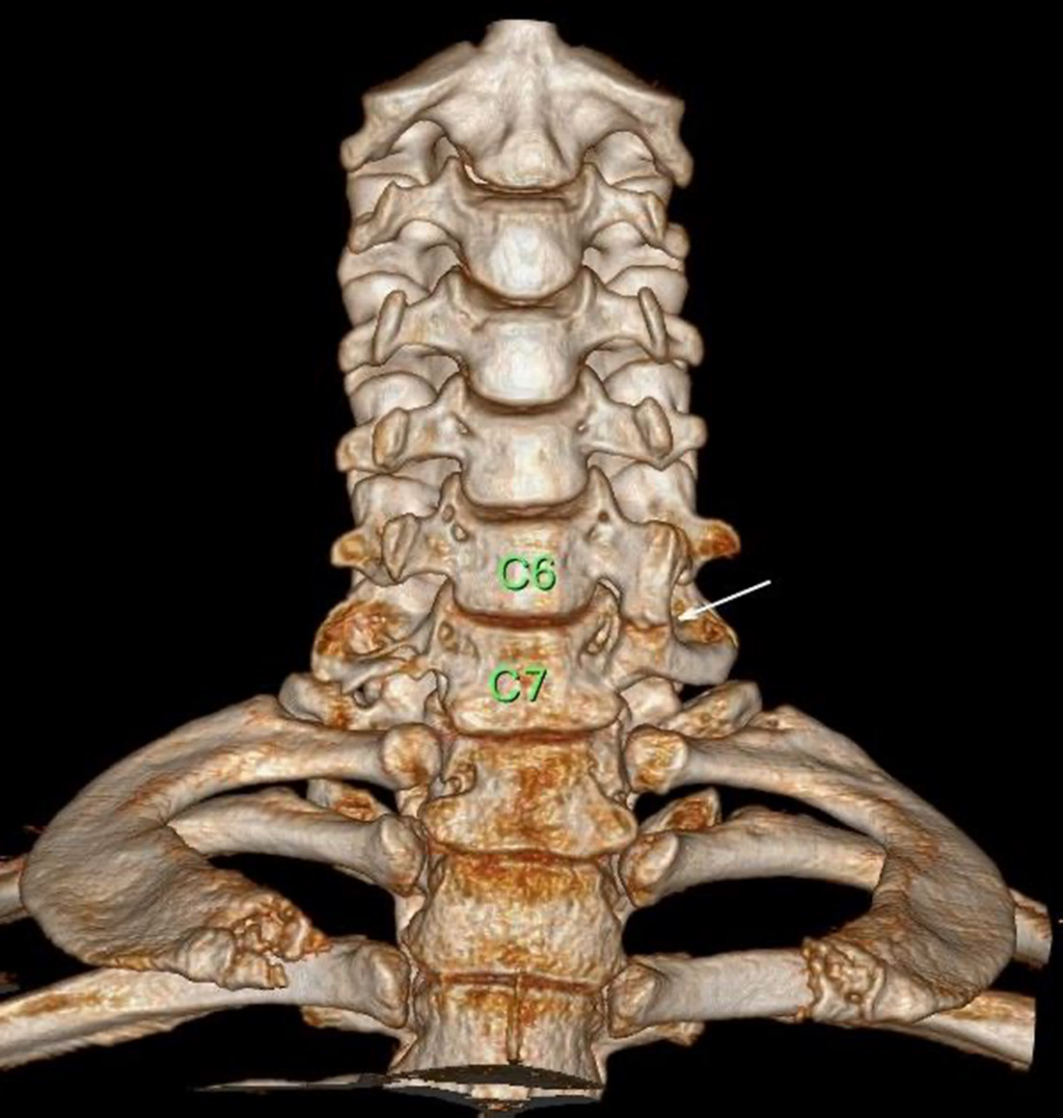
Coronal volume-rendered CT images best delineates the anatomy and clearly shows the accessory articulation between the C6/C7 transverse processes distinguishing it from an acute fracture.

Plain film radiographs were not indicated as part of the initial management but were performed retrospectively for academic reasons in case 2. The plain radiographs showed that there was a bony projection extending anterior to the vertebral bodies with a radiolucent line between the lateral masses of C6/C7 (Figure 2) that corresponded to the variant found on CT (Figure 3). Similar findings could be seen on CT at the C5/C6 level from Case 1 (Figure 4). The variant could easily be confused for a fracture in a trauma setting or osteo-degenerative changes. Osteophytes usually project anterior rather than posterior to the vertebral body margins on lateral film.^3^ The elongation of the anterior tubercle with the accessory articulation was best demonstrated on CT with multi-planar reformations (Figures 3 and 4) and volume-rendered images (Figures 5 and 6). This anomaly is difficult to discern on plain films and is, therefore, more likely to be found incidentally on CT. CT is superior to conventional radiographs with regard to anatomical delineation and is, therefore, the preferred investigation when distinguishing this variant anatomy from fractures and osteo-degenerative changes.

## Conclusion

Accessory articulation of the cervical transverse processes is extremely rare, particularly at the C6/C7 level. In the acute trauma setting, both plain films and CT are of value, with CT being more superior at distinguishing this variant anatomy from fractures and osteo-degenerative changes. These normal variants should be kept in mind when reporting on CT for traumatic injuries to reduce unnecessary immobilization and inappropriate specialist referrals.

## Learning points

- Radiologists should be aware of the normal ossification of the cervical vertebrae. Each cervical vertebra has three ossification centres, the transverse processes are formed by the lateral extension of the centres of the neural arches.
- The costal portion of the transverse process may enlarge and elongate the anterior tubercle to articulate with the vertebrae below giving rise to an unusual variant which may simulate a fracture in an acute trauma setting.
- CT is superior to conventional radiographs with regards to anatomical delineation and is, therefore, the preferred choice at distinguishing variant anatomy from acute fractures.
